# The Potential Use of Matrix Metalloproteinases in Alzheimer’s Disease Therapy

**DOI:** 10.3390/jcm15041555

**Published:** 2026-02-16

**Authors:** Daria Krawczuk, Barbara Mroczko

**Affiliations:** 1 Department of Neurodegeneration Diagnostics, Medical University of Bialystok, Waszyngtona 15A, 15-269 Białystok, Poland; daria.krawczuk@sd.umb.edu.pl; 2 Department of Biochemical Diagnostics, Medical University of Bialystok, Waszyngtona 15A, 15-269 Białystok, Poland

**Keywords:** Alzheimer’s disease, metalloproteinases, amyloid, tau, neuroinflammation

## Abstract

Alzheimer’s disease is the most common cause of dementia and one of the greatest challenges of current medicine. Its pathophysiology is complex, involving β-amyloid deposition, tau hyperphosphorylation, chronic neuroinflammation, and progressive neuronal loss. Despite the introduction of novel therapies, treatment efficacy remains limited, prompting the search for alternative therapeutic targets. One promising area of research focuses on matrix metalloproteinases-proteolytic enzymes involved in tissue remodeling, synaptic plasticity, and inflammatory responses. In the context of AD, MMPs may exert both protective effects, through amyloid degradation, and detrimental effects such as blood–brain barrier disruption and amplification of neuroinflammatory damage. Understanding the dual and context-dependent roles of MMPs may help explain past translational failures and enable the development of more selective, stage-dependent therapeutic strategies. This article is a narrative review summarizing current evidence on the roles of MMPs in AD, with a particular focus on their therapeutic modulation and potential implications for future clinical research. Insights into MMP biology may ultimately guide the design of interventions with improved efficacy and safety for patients with AD.

## 1. Introduction

Alzheimer’s disease (AD) is a leading cause of dementia, accounting for 60–70% of all cases worldwide. Currently, over 50 million people are affected by AD, and this number is expected to rise substantially with ongoing population aging, making AD one of the greatest medical, social, and economic challenges of the 21st century. The increasing prevalence of AD imposes a substantial burden on healthcare systems worldwide, highlighting the urgent need for novel therapeutic strategies [1]. Clinically, the disease is characterized by progressive memory loss, cognitive impairment, and functional decline, while neuropathological hallmarks include extracellular amyloid-β plaques, intracellular neurofibrillary tangles composed of hyperphosphorylated tau protein, chronic neuroinflammation, and widespread neuronal degeneration [1,2]. Despite decades of research, therapeutic progress in AD has been modest. Currently available symptomatic treatments, such as cholinesterase inhibitors and NMDA receptor antagonists, provide only limited benefits. More recently, monoclinal antibodies targeting Aβ have been approved, but their clinical impact remains controversial and associated with safety concerns. These limitations emphasize the urgent need to identify additional molecular targets and pathways contributing to AD pathophysiology [2].

Matrix metalloproteinases (MMPs), a family of zinc-dependent endopeptidases, have recently attracted attention in the context of neurodegenerative diseases. Traditionally recognized for their role in extracellular matrix (ECM) remodeling, MMPs are known to participate in diverse processes within the central nervous system, including synaptic plasticity, neuroinflammation, and a blood–brain barrier (BBB) integrity. Intriguingly, several MMPs have been implicated in Aβ degradation, suggesting a potential protective role. However, dysregulated MMP activity may conversely exacerbate neuronal injury by promoting BBB disruption and amplifying inflammatory responses [3]. The primary objective of this review is to summarize current evidence on the involvement of MMPs in key pathological processes of AD. Secondary objectives include discussing therapeutic strategies targeting MMP activity and highlighting translational challenges. The literature for this narrative review has been identified through searches of PubMed, Web of Science, and Scopus, focusing on publications from the last 10 years. Keywords included “matrix metalloproteinases,” “Alzheimer’s disease,” “amyloid,” “tau,” and “neuroinflammation.” Additional relevant articles were identified through reference screening.

## 2. MMPs in Alzheimer’s Disease

Matrix metalloproteinases are a family of zinc-dependent endopeptidases that play a central role in the degradation and remodeling of the ECM. They are widely secreted and expressed by neurons and glia and participate in diverse physiological processes, including tissue development, wound healing, angiogenesis, and synaptic plasticity. In the central nervous system, MMPs contribute not only to structural remodeling but also to regulation of neuronal signaling, inflammatory responses, and maintenance of the blood–brain barrier. Moreover, MMPs may be secreted into the extracellular space in case of brain damage or during neuroinflammation and neurodegenerative processes by the activated microglia and astrocytes, which is called reactive gliosis [4].

MMPs are classified into several subgroups based on substrate specificity and structural features, including collagenases, gelatinases, stromelysins, matrilysins, and membrane-type MMPs (MT-MMPs). MMPs are made up of the following domains: signal peptide, propeptide, catalytic, hinge region, and hemopexin-like domain. They are synthesized as inactive precursors (pro-MMPs), which are activated into functional proteases through a mechanism called the cysteine switch [4]. Their activity is tightly regulated at multiple levels: transcriptional control, activation of latent proenzymes (zymogens), and inhibition by endogenous tissue inhibitors of metalloproteinases (TIMPs) as well as by other non-specific inhibitors such as α2-macroglobulin, or plasma inhibitor. This regulation is crucial, as imbalances in MMP activity can lead to excessive ECM degradation, inflammation or tissue damage [5].

The involvement of MMPs in AD has gained increasing attention, as these enzymes are positioned at the crossroads of amyloid metabolism, tau pathology, neuroinflammation, and blood–brain barrier integrity. Their actions appear to be context-dependent, with evidence for both neuroprotective and neurotoxic roles.

### 2.1. Amyloid Metabolism

MMPs play a central role in amyloid beta (Aβ) metabolism, influencing both its clearance and accumulation. Particularly MMP-2 and MMP-9 have been shown to sequentially degrade Aβ proteolytically, producing highly soluble, non-toxic, and non-fibrillogenic C-terminally truncated fragments, typically found in cerebrospinal fluid (CSF) [6,7,8]. The predominant Aβ peptides (Aβ1-42 and Aβ1-40) undergo sequential proteolytic degradation by MMP-2 and MMP-9, generating three principal fragments: Aβ1-34, Aβ1-30 and Aβ1-16. Among these, Aβ1-16 exhibits high solubility and is cleared from the extracellular matrix. Furthermore, activated MMP-9 exhibits low efficiency in this hydrolytic cascade, resulting in limited conversion of intermediates into Aβ1-16. In contrast, activated MMP-2 efficiently hydrolyzes intact Aβ1-40 and Aβ1-42, producing the soluble 16-aminoacid fragment suitable for clearance. The concerted activity of MMP-9 and MMP-2 is therefore proposed to promote amyloid plaque disaggregation and the hydrolysis of amyloidogenic species [6].

Additionally, the activator of MMP-2, MT1-MMP can also degrade soluble and fibrillar Aβ1-40 and Aβ1-42 in a time-dependent manner [9]. MMP-2 is involved in the degradation of Aβ peptides and their plaques. It can cleave Aβ, facilitating its clearance, which suggests that it could play a protective role against amyloid toxicity in the brain [10]. MMP-9 activity is associated with Aβ levels, indicating that increased MMP-9 in Alzheimer’s disease might have both beneficial and harmful impacts, depending on the local concentration and specific circumstances. Nevertheless, overly high MMP-9 activity can cause neurovascular impairment, worsening the neurodegeneration linked to AD [11].

The balance between MMP-9 and TIMP-1 is also crucial, as it has been shown that impaired MMP-9/TIMP-1 ratio can lead to either excessive degradation of the ECM or insufficient remodeling, contributing to the neurodegenerative process. Such disruptions can promote neurodegeneration by impeding neuronal repair and intensifying neuroinflammation. Additionally, TIMP-1 plays a role in controlling inflammation through its regulation of MMP activity [12]. It has been demonstrated that an elevated MMP-9/TIMP-1 ratio precedes the emergence of new gadolinium-enhancing lesions, indicating that the balance between these molecules may serve as a marker of blood–brain barrier permeability to pro-inflammatory cells and that peripheral blood measurements can reliably reflect neuroinflammatory activity [13]. Therefore, MMP-mediated amyloid metabolism is a double-edged process: while certain MMPs contribute to neuroprotection through Aβ clearance, unregulated activity can produce collateral damage. Understanding the balance between these opposing effects is essential for leveraging MMPs in therapeutic strategies for AD.

Additionally, by modulating BACE1 activity, MMP-13 may substantially impact Aβ production levels. For example, inhibiting MMP-13 in transgenic mouse models of Alzheimer’s disease has resulted in decreased amyloid pathology and enhanced cognitive function, underscoring its potential as a therapeutic target [14].

MT5-MMP activity is found to exert pro-amyloidogenic properties because CTF-η can be processed by β-secretase and γ-secretase to yield Aβ [15]. It has been reported that mutations in specific domains of MT5-MMP can significantly impact the accumulation of toxic APP metabolites, supporting the hypothesis that the proteolytic action of MT5-MMP is engaged in AD pathology. Furthermore, MT5-MMP is capable of cleaving APP in cell models, consistent with its pro-amyloidogenic role in the disease process [16]. In another study it has been demonstrated that MT5-MMP deficiency resulted in reduced amyloid pathology and cognitive decline in 5xFAD mice [17]. By regulating the pathways through which APP is processed, MT5-MMP could impact the overall amyloidogenic pathway, positioning it as a critical target for controlling APP metabolism and the accumulation of neurotoxic Aβ [18]. Thus, combining MT5-MMP modulation with other therapeutic strategies targeting amyloid pathology or neuroinflammatory pathways may yield synergistic effects. This multifaceted approach could enhance the therapeutic efficacy of treatments in AD, particularly given the interplay between amyloid accumulation and MMP activity.

### 2.2. Tau Pathology

Tau protein, a microtubule-associated protein primarily expressed in neurons, plays a key role in maintaining cytoskeletal stability and axonal transport. In AD, tau becomes hyperphosphorylated, detaches from microtubules, and aggregates into neurofibrillary tangles (NFTs), contributing to synaptic dysfunction and neuronal death. The interplay between MMPs and tau pathology in AD presents significant opportunities for therapeutic strategies. Emerging evidence suggests that MMPs can modulate tau pathology through direct and indirect mechanisms [19].

MMP-2 has been detected in certain neurons containing NFTs and in dystrophic neurites, where it co-localized with hyperphosphorylated tau, as demonstrated through double-labeling immunofluorescence and confocal microscopy, suggesting a close association between MMP-2 and hyperphosphorylated tau in AD. Terni et al. showed elevated expression levels of MMP2 at early and middle stages of AD-related pathology compared with middle-aged cases with no AD-related pathology [20]. Therefore, the increased expression of MMP-2 during the early and middle stages of AD-related pathology might represent an additional mechanism that facilitates tau fibrillization. Additionally, the inability of MMP-2 to break down hyperphosphorylated tau within paired helical filaments could further promote the buildup of tau aggregates [20]. An imbalance favoring MMP-2 activity may disrupt tau metabolism, compounding the effects of tau-induced neurotoxicity [21]. Furthermore, MMP-2 may facilitate processes that enhance the formation of tau oligomers that could promote their spread across neuronal networks, as tau pathology is known to propagate in a prion-like manner [22].

MMP-9 has been shown to cleave tau at both the N- and C-terminal regions, facilitating the release of tau fragments with increased propensity for aggregation. Furthermore, research indicates that the presence of elevated MMP-9 levels correlates with the progression of tau-related pathology, as observed in post-mortem brain samples from AD patients [23]. While MMP-9 may facilitate tau toxicity through cleavage and subsequent aggregation, MMP-2’s role appears more complex, possibly linking to early pathological events rather than direct tau cleavage [6,20].

### 2.3. Neuroinflammation

Neuroinflammation is a hallmark of AD, characterized by a chronic activation of microglia and astrocytes, release of pro-inflammatory cytokines, and recruitment of peripheral immune cells. MMPs are central regulators of these processes, influencing both the initiation and amplification of inflammatory processes [24].

Elevated levels of inflammatory cytokines, such as interleukin-1 beta (IL-1β), are known to enhance MMP-2 expression, leading to an increased matrix degradation and potential neuronal damage [25]. Moreover, the activation of MMP-2 correlates with increased neuroinflammatory processes, which may exacerbate neuronal damage and contribute to the neurodegenerative pathways observed in AD [26]. Activated MMPs, especially MMP-9 and MMP-2, degrade collagen and laminin within the basement membrane, as well as break down tight junction proteins. This process compromises the integrity of BBB and promotes the entry of immune cells and inflammatory mediators into the central nervous system, thereby intensifying neuroinflammation [27].

Crocker et al. observed that microglia, rather than astrocytes, are the primary source of MMP-9 following stimulation with pro-inflammatory molecules such as lipopolysaccharides or tumor necrosis factor α (TNFα) [28]. In contrast, astrocytes expressed MMP-2, -11, and -14 and exhibited a modest induction of MMP-3 in response to IL-1β [28]. Additionally, elevated levels of MMP-13 may exacerbate inflammatory responses in the brain, potentially leading to neuronal death and accelerated neurodegeneration [29]. Furthermore, Piłat et al. reported that MT5-MMP promotes neuroinflammation in primary neuronal cultures derived from 5xFAD mice, a model of amyloidosis [30]. This study indicates that MT5-MMP may contribute to inflammation driven by Aβ and could exacerbate AD pathology. Targeting MT5-MMP could thus reduce both amyloid levels and neuroinflammatory responses [30].

Thus, selective inhibition of pro-inflammatory MMPs could reduce glial activation and cytokine-mediated neuronal injury without interfering protective roles such as Aβ clearance. Targeting MMPs in combination with anti-inflammatory agents may offer synergistic benefits, providing a more comprehensive approach to mitigate chronic neuroinflammation in AD.

### 2.4. Blood–Brain Barrier Dysfunction

BBB integrity is essential for maintaining central nervous system homeostasis. In AD, its disruption is increasingly recognized as an early pathological event contributing to neuroinflammation, neuronal injury, and amyloid accumulation. MMPs, particularly MMP-2, MMP-9, and MMP-13, play central roles in BBB remodeling through degradation of tight junction proteins such as occludin (OCLN), claudin-5 (CLDN5), and zonula occludens-1 (ZO-1) [31].

MMP-2 activity is particularly linked to vascular damage in the brain because of its ability to break down tight junction proteins essential for maintaining the structural integrity of BBB. When the BBB is compromised due to this dysregulation, harmful substances can enter the central nervous system, leading to neuroinflammation that plays a role in the progression of AD [32]. Additionally, MMP-2 has been identified in astrocytes and neurons in the AD brain, where it is localized around amyloid plaques. This localization suggests that the enzyme may participate in the response to neurodegenerative changes, potentially influencing the integrity of BBB [33]. Dysregulation of MMP-2 can, in turn, lead to BBB breakdown, allowing for further complications such as increased neuroinflammation and deposition of neurotoxic substances [34].

MMP-9 has been demonstrated to break down the connections between endothelial cells. This disruption allows amyloid proteins to pass through the blood vessel walls more easily, contributing to inflammation in the nervous system [35]. The relationship between MMP-9 and the apolipoprotein E (APOE) ε4 allele, a significant genetic risk factor for AD, is particularly noteworthy. APOE4 has been shown to enhance MMP-9 activation, thereby impairing BBB integrity and facilitating Aβ accumulation [36].

MMP-13 also contributes to BBB dysfunction by cleaving extracellular matrix proteins and promoting inflammatory signaling via NF-κB activation. MMP-13 is involved in the degradation of various ECM components. Dysregulation of MMP-13 can lead to a breakdown of this barrier, allowing neurotoxic elements, including Aβ, to penetrate the brain more readily [37].

In conclusion, MMPs significantly influence BBB integrity and contribute to the pathogenic processes underlying AD. Targeting MMP activity through inhibition or modulation represents a promising therapeutic strategy for ameliorating BBB dysfunction and mitigating neuroinflammation in AD. While promising results from preclinical studies are encouraging, further research is essential to translate these findings into effective clinical therapies [38]. Table 1 summarizes the role of selected MMPs in AD.

## 3. Therapeutic Implications of MMPs Modulation

The dual nature of MMPs in AD presents both a challenge and an opportunity for therapeutic interventions. Selective inhibition of neurotoxic MMPs (e.g., MMP-3, MMP-9, MT5-MMP) while preserving or enhancing the activity of protective enzymes (e.g., MMP-2) could yield neuroprotective benefits. Early attempts using broad-spectrum MMP inhibitors were limited by toxicity and poor selectivity, but recent advances in structure-based drug design and RNA-based modulation have renewed interest in targeted approaches [48].

### 3.1. Small-Molecule Inhibitors

The use of small-molecule inhibitors targeting MMPs has gained attention as a potential therapeutic strategy in AD due to the involvement of these enzymes in various pathological processes underlying the disease. These molecule inhibitors aim to modulate the degradation of the extracellular matrix and regulate the inflammatory processes mediated by MMPs [49].

The role of mitogen-activated protein kinases (MAPKs) in the modulation of MMPs, particularly MMP-9, in the context of AD is significant because these intracellular signaling pathways regulate various cellular processes critical to neuroinflammation and neurodegeneration [50]. MAPK pathways, including extracellular signal-regulated kinase (ERK), c-Jun N-terminal kinase (JNK), and p38 MAPK, interact with the regulation of MMP gene expression and activity, influencing the pathology of AD. MMP-9 expression is notably regulated by MAPK signaling pathways. Specific studies have demonstrated that the p38 MAPK pathway is critical for the induction of MMP-9 in various cell types, including monocytes and vascular smooth muscle cells [49,50]. Blocking ERK or p38 MAPK has been observed to effectively reduce MMP-9 expression, highlighting a potential therapeutic angle in alleviating MMP-9’s contribution to neurodegenerative processes in AD [51]. The interplay between MAPK signaling and MMP-9 suggests potential therapeutic implications, as pharmacological targeting of these pathways may help regulate the detrimental effects of MMP-9 in the pathology of AD. Overall, the modulation of MMP-9 through MAPK signaling pathways elucidates an intricate mechanism contributing to the neuroinflammatory processes that is typical in AD.

Batimastat (also known as BB-94) is a potent MMP inhibitor that has been studied for its potential therapeutic effects in AD. Its mechanism of action involves competitive reversible inhibition of MMPs, including α-secretase, which plays a crucial role in the processing of APP and the generation of Aβ peptides, responsible for neuronal toxicity in AD [52]. By inhibiting α-secretase, batimastat reduces the production of Aβ peptides, thereby addressing one of the critical pathological features of AD. This effect on APP processing suggests that batimastat could play a role in mitigating Aβ accumulation and subsequent neurotoxicity [53]. However, it should be noted that its clinical use in AD treatment has been limited by noted side effects, including unexpected alterations in cognition and behavior observed in preclinical studies [54]. Identifying optimal dosing and reducing side effects will be crucial as research progresses.

Epigallocatechin gallate (EGCG) is the most abundant catechin found in green tea and is noted for its health benefits, which include anti-inflammatory and antioxidant properties. Its potential role in AD has garnered significant interest, particularly concerning its interactions with MMPs and the pathways involved in neurodegeneration. EGCG has been shown to inhibit the activity of MMP-2 and MMP-9. Specifically, EGCG has been reported to suppress the mRNA expression of MMP-2, contributing to its anti-inflammatory effects [55]. Moreover, by modulating the activity of signaling pathways such as the MAPK pathway, EGCG can effectively diminish MMP expression and improve neuronal survival [56]. It has been demonstrated to reduce the phosphorylation of ERK and p38 MAPK, which are critical for the expression of pro-inflammatory mediators and MMPs [57]. This modulation may potentially reduce neuroinflammation and protect against synaptic dysfunction linked to tau pathology in AD. The promising findings regarding EGCG’s efficacy in the inhibition of MMPs and the amelioration of neuroinflammatory responses underscore its potential as a therapeutic agent for AD. However, clinical investigations are needed to establish optimal dosing, bioavailability, and specific effects on cognitive outcomes in AD patients. The potential need for clinical trials examining the long-term effects of EGCG, particularly when combined with other anti-inflammatory agents, presents an area for future research [55].

Doxycycline, a semi-synthetic derivative of tetracycline, has emerged as a candidate for repurposing in the treatment of AD due to its anti-inflammatory and neuroprotective properties [58]. Doxycycline has been shown to effectively inhibit MMP-2 and MMP-9 [58,59]. By inhibiting these enzymes, doxycycline may help mitigate the inflammatory response and protect against neurodegeneration. Studies indicate that doxycycline-mediated inhibition of MMPs may also reduce the pathological processing of Aβ peptides, contributing to reduced amyloid plaque formation [58]. A pilot study examining the effect of doxycycline in treating amyloidosis indicated that it could ameliorate conditions associated with elevated MMP activity, exhibiting its potential for application beyond traditional antibiotic roles. Additionally, the adjunctive use of doxycycline, in combination with therapies targeting amyloid aggregation, suggests a multi-targeted approach for therapeutic intervention in AD [59]. Doxycycline is generally well-tolerated in clinical settings and has a favorable safety profile, which further supports its potential as a long-term treatment option for AD. Its effects on maintaining gut flora and minimal antibiotic resistance make it a suitable candidate for further exploration and potential application in chronic neurodegenerative conditions [60]. In conclusion, doxycycline represents a multifaced therapeutic agent in the management of AD through its roles as an MMP inhibitor, an anti-inflammatory agent, and a neuroprotective compound. Continued research, including randomized controlled trials and mechanistic studies, will be crucial in confirming its efficacy and optimizing treatment regimens involving doxycycline [58,59,60].

**Figure 1:**
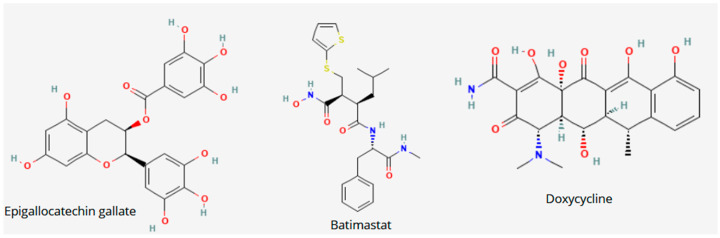
Chemical structures of epigallocatechin gallate, batimastat and doxycycline.

Each of these small-molecule inhibitors exemplifies the importance of targeting MMPs and their regulatory environments to mitigate the effects of AD. Ongoing research aims to better understand their specific roles, optimal dosing schedules, and potential side effects, as well as their effectiveness in combination therapies. Future studies will need to clarify how these inhibitors can be integrated into existing treatment paradigms for AD [61]. Figure 1 presents the chemical structures of presented inhibitors.

### 3.2. Gene Silencing Tools to Downregulate MMP Expression

Gene silencing therapy, particularly utilizing small interfering RNA (siRNA) technology, has emerged as a promising strategy for targeting MMPs in the context of AD [62]. Small interfering RNA (siRNA) represents a promising approach to regulate MMPs in the context of AD therapy. The modulating effects of siRNA on MMP expression can play a critical role in alleviating neuroinflammation, preserving cognitive function, and potentially modifying the disease’s progression. siRNA technology operates by delivering small, double-stranded RNA molecules that selectively bind to and promote the degradation of specific mRNA transcripts through sequence complementarity. This mechanism leads to the suppression of gene expression after transcription, thereby decreasing the production of proteins involved in disease-related pathways in AD, like MMPs [63]. Several studies have successfully employed siRNA techniques to downregulate MMP expression. For instance, inhibition of MMP-9 expression using siRNA has demonstrated significant reductions in gastric adenocarcinoma cell growth and invasion, indicating that similar strategies could be leveraged to alter MMPs activity in neurons affected by AD [64]. siRNA targeting MMPs has the potential to reduce neuroinflammation in AD models. The MAPK and NF-κB pathways, which are activated during neuroinflammatory responses, are key mechanisms through which siRNA-mediated MMP silencing can exert beneficial effects. For example, the silencing of MMP-2 has been shown to inhibit inflammatory responses in other contexts, suggesting potentially analogous effects in AD [65]. The application of siRNA to specifically target MMPs in AD therapy may involve using delivery systems that can effectively transfect neuronal cells. Advances in nanoparticle technology and viral vectors provide promising avenues for efficiently delivering siRNA to the brain, ensuring targeted MMP gene silencing and reducing potential off-target effects. For instance, synthetic and biodegradable nanoparticles have been investigated for their ability to deliver siRNA effectively to MMP-rich environments, enabling localized action against pathological processes in AD [66]. Thus, siRNA serves as a potent therapeutic tool for specifically modulating MMP expression in AD. By targeting MMPs, siRNA can potentially reduce neuroinflammation, inhibit amyloid and tau-related pathologies, and contribute to the neuroprotective landscape necessary for combating AD. Continued development and evaluation of siRNA strategies will be essential to harness their full therapeutic potential in clinical settings.

Small hairpin RNA (shRNA) has emerged as a powerful tool in the therapeutic landscape for AD, particularly in its application for modulating the expression of MMPs [67]. The effectiveness of shRNA in modulating MMPs is particularly relevant to controlling pathological processes in AD. By deploying shRNA against MMPs, research aims not only to silence the expression of these enzymes but also to regulate the downstream signaling cascades (e.g., MAPK and NF-κB pathways) that mediate inflammatory responses, further potentiating neuroprotective effects [68]. The utility of shRNA targeting MMPs in preclinical models of AD has shown encouraging results. Studies have highlighted the successful application of lentiviral vectors expressing shRNAs to silence specific MMP genes, leading to reduced levels of inflammatory cytokines and improved neuronal viability in animal models [69]. In summary, shRNA represents a novel and potentially effective strategy for modulating MMPs in AD therapy. Targeted silencing of MMPs through shRNA holds promise for reducing neuroinflammation and mitigating the progression of AD, necessitating further research to establish practical applications and optimize therapeutic protocols.

The application of CRISPR technology in targeting MMPs for the treatment of AD represents a promising frontier in gene therapy. CRISPR/Cas9 has shown potential for precise gene editing, allowing researchers to specifically disrupt or modify the expression of MMP genes, such as MMP-2 and MMP-9, which are often upregulated in AD patients. The application of CRISPR/Cas9 may reduce MMP-9 expression through the delivery of guide RNA (gRNA) that directs Cas9 to the MMP-9 gene, resulting in targeted gene knockout that alleviates neuroinflammation and amyloid pathology [70]. Efficient delivery of the CRISPR components (Cas9 and gRNA) is essential for effective gene editing. Viral vectors such as adeno-associated viruses (AAVs) are often used due to their ability to transduce neuronal cells successfully. Strategies incorporating nanoparticles and other delivery methods can enhance cellular uptake of CRISPR components and improve gene-editing efficiency in vivo [71]. While the potential of CRISPR/Cas9 in targeting MMPs for AD is vast, several challenges remain. Off-target effects and delivery efficiency must be carefully evaluated to ensure that genetic modifications do not inadvertently affect other genes or pathways. Additionally, regulatory obstacles and long-term impacts of gene editing raise important ethical considerations in the translation of CRISPR technology to clinical settings [72]. In summary, CRISPR technology presents a groundbreaking approach to modulate MMP expression in Alzheimer’s disease therapy. By targeting MMPs responsible for neuroinflammation and amyloid pathology, researchers potentially pave the way for innovative treatment options that could significantly halt or modify the progression of AD. Further investigations, including preclinical studies and optimized delivery methods, will be critical to advance this therapeutic strategy.

### 3.3. Repetitive Transcranial Magnetic Stimulation

Repetitive transcranial magnetic stimulation (rTMS) has emerged as a non-invasive treatment method for AD, showing promise for improving cognitive function and modulating neuroinflammatory processes. In recent years, research has begun to explore the relationship between rTMS, MMPs, and AD [73].

The study by Cirillo et al. indicated that long-term rTMS applications could lead to decreases in plasma levels of MMPs, particularly in patients with mild cognitive impairment (MCI) [74]. This suggests that rTMS may exert a neuromodulatory effect on the expression of MMP-9, which is associated with neuroinflammation and cognitive impairment in AD. The study hypothesizes that the reduction in circulating MMPs may correlate with improvements in cognitive abilities following rTMS treatment [74]. In another study, authors discussed how rTMS could positively affect neurotrophic factors, counteracting amyloid and tau accumulation, and mitigating neuroinflammation, which are all processes influenced by MMP activity. The ability of rTMS to attenuate the inflammatory responses associated with elevated MMP-9 levels may contribute to restoring cognitive functions and improving outcomes in AD patients [75]. Fulopova et al. highlighted that rTMS increases synaptic plasticity of neurons in an APP/PS1 mouse model, which is used to study amyloidosis and the pathological features of AD [76]. This effect is significant as MMPs are known to play important roles in synaptic remodeling. By utilizing rTMS to enhance synaptic plasticity, there may be a complementary modulation of MMP activity, promoting brain health and function during the progression of AD [76]. Thus, rTMS emerges as a potential therapeutic strategy that interacts with MMP pathways in AD. By modulating MMP expression and influencing neuroplasticity and neuroinflammatory responses, rTMS may offer cognitive benefits and slow the progression of AD. Continued research is essential to explore the underlying mechanisms of rTMS in relation to MMPs and to refine treatment strategies for optimal patient outcomes.

## 4. Discussion and Future Directions

### 4.1. Translational and Clinical Implications

MMPs represent a critical but complex component of AD pathophysiology. Acting at the interface of amyloid metabolism, tau pathology, neuroinflammation and blood–brain barrier integrity, they exert both protective and deleterious effects. Specific MMPs, such as MMP-2 and MMP-9, contribute to amyloid clearance while others, including MMP-3 and MMP-12, amplify neuroinflammatory cascades and neuronal injury [64].

Despite growing evidence for the involvement of MMPs in AD, several key challenges remain. First, the dual and context-dependent role of MMP activity complicates their therapeutic targeting. Protective roles, such as Aβ clearance, coexists with detrimental effects, including BBB disruption and amplification of neuroinflammation. Disentangling these processing will require deeper mechanistic insights and stage-specific analyses of AD [77]. Second, most current data on MMPs in AD arise from preclinical models, and translation into human studies remains limited. Large-scale clinical studies investigating MMP levels in CSF and blood are necessary to validate their potential as biomarkers of disease progression and therapeutic response. In parallel, advanced imaging methods and single-cell transcriptomic approaches may help clarify the cellular and spatial regulation of MMP activity in the diseased brain [78]. Third, therapeutic innovation depends on the development of highly selective modulators of MMPs. The design of small molecules, monoclonal antibodies, or RNA-based tools targeting individual MMPs must overcome the challenge of poor selectivity observed with broad-spectrum inhibitors. Strategies combining MMP modulation with existing anti-amyloid and anti-tau therapies may provide synergistic benefits. Finally, the future of MMP research in AD likely lies in the integration of biomarker-driven, precision medicine approaches. Stratifying patients based on inflammatory activity, vascular dysfunction, or amyloid burden may help adjust MMP-targeted interventions to those most likely to benefit [79].

It is crucial to understand that although MMPs have potential as therapeutic targets, their dysregulation or overexpression can cause harmful effects such as increased blood–brain barrier permeability and neuroinflammation, worsening the disease. Thus, a balanced approach is necessary, and strategies aimed at specifically enhancing safe MMP activity without inducing adverse responses must be prioritized [80].

The development of targeted MMP modulators holds significant potential for future Alzheimer’s disease therapies, emphasizing the need for continued research in this complex area. Although direct clinical applications remain limited, understanding MMP biology may inform the timing, stratification, and combination of future therapeutic and supportive strategies. While this review focuses on the molecular and translational aspects of MMPs in AD, future studies could consider how these findings integrate with broader clinical and supportive care strategies, including emerging digital health tools and assistive technologies.

### 4.2. Limitations

This review has some limitations. First, the studies included are heterogeneous, ranging from in vitro experiments and animal models to post-mortem and limited clinical data, which may reduce the comparability of findings. Second, the dual and context-dependent roles of MMPs, combined with the lack of selective inhibitors, complicate the translation of experimental results into therapeutic strategies. Third, the translational applicability of findings from preclinical studies to human AD remains uncertain. Finally, as a narrative review, this article does not follow a systematic methodology, and the selection of literature may be influenced by author discretion.

## 5. Conclusions

In conclusion, MMPs play pivotal and context-dependent roles in AD, influencing amyloid metabolism, tau pathology, neuroinflammation, and blood–brain barrier integrity. The dual nature of MMPs—both protective and detrimental depending on the specific enzyme, stage of disease, and cellular context—highlights the challenges of designing effective therapeutic interventions. Understanding these complex mechanisms is essential for the rational design of selective MMP modulators that can maximize neuroprotective effects while minimizing potential adverse consequences. Although direct clinical applications remain limited, insights into MMP biology and the properties of different inhibitors may guide future translational research, inform the selection of appropriate preclinical models, and support the development of more precise, stage-specific, and effective interventions for patients with AD. Future studies integrating mechanistic, biomarker, and clinical data are warranted to bridge the gap between experimental findings and therapeutic implementation, ultimately advancing personalized approaches to AD management.

## Figures and Tables

**Table 1:** Selected metalloproteinases involved in Alzheimer’s disease pathophysiology.

MMP	Role	Reference
**MMP-2**	Involved in the degradation of Aβ peptides and their plaques. Can influence the phosphorylation status of tau, thereby affecting its propensity to aggregate. Could modulate tau phosphorylation and aggregation pathways, which are critical in the development of neurofibrillary tangles that are associated with cognitive decline in AD. Increased activity can lead to BBB disruption, promoting neuroinflammation.	[20,26,39]
**MMP-3**	Secreted by astrocytes and microglia in response to inflammatory stimuli. May play a role in reducing Aβ levels while concurrently contributing to increased neuroinflammation, which can cause neuronal damage. Increased levels have also been correlated with cognitive impairment.	[40,41]
**MMP-9**	Can degrade insoluble amyloid fibrils and is thought to contribute to the clearance of Aβ from the brain. Activity correlates with Aβ levels, suggesting that its upregulation in AD could have both protective and detrimental effects, depending on local concentrations and contexts. Excessive activity may lead to neurovascular dysfunction, exacerbating the neurodegenerative process associated with AD. Disrupts endothelial cell junctions, facilitating amyloid transcytosis and promoting neuroinflammation.	[11,35]
**MMP-12**	Can be activated by Aβ peptides, contributing to the degradation of amyloid plaques. Elevated levels in the brains of individuals with AD have been associated with increased neuroinflammation. Its overexpression can lead to detrimental effects, such as disrupting the integrity of BBB and enhancing neuroinflammation. Polymorphisms in the *mmp12* gene have been correlated with increased AD risk.	[42,43,44]
**MMP 13**	Has been found to influence APP processing. Involved in the regulation of BACE1, which generates Aβ peptides from APP. Elevated levels are found in the brains of AD patients, correlating with increased neuroinflammation.	[45,46,47]

## Data Availability

No new data were created or analyzed in this study.
